# Impact of Combined Theory-Based Intervention on Psychological Effects and Physical Activity among Chinese Adolescents

**DOI:** 10.3390/ijerph17093026

**Published:** 2020-04-27

**Authors:** Yao Zhang, Yi Yin, Jianxiu Liu, Ming Yang, Zeshi Liu, Xindong Ma

**Affiliations:** Division of Sports Science & Physical Education, Tsinghua University, Qinghuayuan Street, Haidian District, Beijing 100084, China; yao-zhan19@mails.tsinghua.edu.cn (Y.Z.); 18601117163@163.com (Y.Y.); liujianx17@mails.tsinghua.edu.cn (J.L.); yangming13078@163.com (M.Y.); 15110033155@139.com (Z.L.)

**Keywords:** adolescents, physical activity intervention, extended theory of planned behavior, psychological construct improvement, health-related behavior modification

## Abstract

Purpose: The current study was intended to assess the effect of a facilitated behavioral intervention based on the extended theory of planned behavior (TPB) on psychological constructs and physical activity among adolescents over a period of eight weeks. Methods: Students (*n* = 51, 12 ± 0.3 years of age) in the seventh grade at a junior middle school in China were randomly assigned to two groups: the intervention group (*n* = 24) and the control group (*n* = 27). Both groups were pre- and post-tested with the related psychological constructs of the extended TPB, along with behavioral measures of the Physical Activity Scale and ActiGraph accelerometer (model wGT3X-BT). The intervention group took part in 45 min classes once per week for 8 weeks, including five indoor theoretical courses and three outdoor basketball matches. The control group was not required to make any change to their normal school day. Also, 2 × 2 repeated measures analysis of variance (ANOVA) was conducted to compare the differences between the two groups, and then *t*-test was employed to compare the independent and paired differences. Results: Significant increases in pre–post subjective norms (SN) (*p* = 0.041, Cohen’s d = 0.62), perceived behavior control (PBC) (*p* = 0.023, Cohen’s d = 0.72), exercise intention (EI) (*p* = 0.043, Cohen’s d = 0.61), and self-efficacy (SE) (*p* = 0.035, Cohen’s d = 1.36) were observed in the intervention group. In addition, participants in the intervention group increased their exercise frequency (*p* < 0.001, Cohen’s d = 1.25) and intensity (*p* = 0.028, Cohen’s d = 0.68), especially their time spent on light intensity physical activity (light-PA%; *p* = 0.031, Cohen’s d = 0.68), and their percentage of sedentary time (SB%) was also reduced (from 68% ± 10% to 58% ± 7%, *p* < 0.001, Cohen’s d = 1.17). Furthermore, the intervention group showed significantly better performance in PBC (*p* = 0.032, Cohen’s d = 0.62), EI (*p* < 0.001, Cohen’s d = 1.32), SE (*p* < 0.001, Cohen’s d = 1.15), SB% (*p* < 0.001, Cohen’s d = 1.22), light-PA% (*p* < 0.001, Cohen’s d = 1.12), and total physical activity (TPA) (*p* = 0.015, Cohen’s d = 0.72) compared to the control group at the post-test. No significant pre post differences were observed for any psychological or behavioral variables in the control group, except for exercise frequency, but the values were still lower than those in the intervention group after the 8-week intervention (3.70 ± 0.72 versus 3.92 ± 0.83). Conclusion: The combined theory-based intervention was effective at improving psychological constructs and physical activity among seventh-grade adolescents in 8 weeks.

## 1. Introduction

Insufficient physical activity (PA) has been ranked as the fourth highest risk factor globally for premature mortality by the World Health Organization (WHO) [1]. Considerable evidence indicates that inadequate physical activity has an adverse effect on physiological and psychological health [2,3] and can potentially trigger chronic diseases such as diabetes [4] and obesity [5] among children and teenagers. Moreover, as physical inactivity during childhood tends to track into adulthood [6], providing adolescents with adequate physical activity has been a worldwide public health priority.

According to nationwide surveys, the level of physical activity and health status of Chinese school-aged youths showed a downward trend from around 22.7% in 2010 to only about 8.9% in 2014 [7]. Although many policies have been put in place to improve adolescents’ PA level, only less than one-third of Chinese adolescents could meet the recommended level of 60 min of moderate and vigorous physical activity (MVPA) in 2016 [7]. As the physical activity level of teenagers can continue to decrease with age, which could be well above the recommended MVPA at 9 years but only 17% by 15 years [8], it is very important to conduct effective interventions to improve the physical activity of Chinese adolescents.

Schools are generally considered to be important places for adolescents to improve their physical activity and keep healthy [9] because almost all adolescents spend extended periods of time at school [10], and schools have many resources including sport equipment, facilities, related curricula, and qualified physical education (PE) teachers to promote PA [11]. According to an American study, the proportion of time middle school students spend on MVPA can be increased to 58.7% by changing the content and teaching methods of physical education (PE) [12]. A number of studies [13,14,15] also indicate that school-based interventions can play an effective role in facilitating students’ physical activity level and increasing their psychological well-being. Moreover, previous studies [13,14,16,17,18] showed that multicomponent interventions based on appropriate theory were more likely to succeed in improving physical activity, with a longer duration of effectiveness than those without an explicit theory basis. Such specific interventions are helpful to develop good exercise habits and transform exercise intention to actual PA behavior more strongly. A previous study [19] also recommended combining subjective and objective ways of monitoring physical activity behavior considering the accuracy of measurement.

The theory of planned behavior (TPB) [20], with exercise attitude (EA), subjective norms (SN), perceived behavioral control (PBC), and exercise intention (EI), as well as social cognitive theory (SCT) [21], with core concepts of self-efficacy (SE) and outcome expectancy (OE), are classic health-promotion models with internal and external factors forming the behavior process. Constructs of TPB and SCT have been verified to be powerful predictors of physical activity behavior in many studies [22,23,24,25]. Recently, foreign studies on teenagers’ physical activity transformed from theoretical prediction to practical theory-based intervention, even if some results of those interventions showed inconsistency [26,27,28]. For example, Dishman [27] found that theory-based interventions among adolescent girls could directly impact their self-efficacy and partially influence PA, while intervention outcomes in Lee’s study [28] showed nonsignificant effects on adolescents’ exercise self-efficacy and PA levels. However, China lacks a relatively effective intervention study with theory employed as the basis, especially for adolescents [29]. Given the advanced studies abroad and the limited study of effective interventions targeting improved spontaneous physical activity among Chinese adolescents, the primary aim of this study was to evaluate the effects of a multicomponent intervention based on the extended TPB, including self-efficacy and outcome expectancy, on promoting psychological constructs and physical activity among Chinese adolescents combining subjective and objective measurements. The hypothesis of the study was that the specific intervention would lead to improved PA-related psychological effects, such as SE, EA, and EI, and would affect PA behavioral factors such as energy expenditure, PA intensity, and sedentary behavior.

## 2. Materials and Methods

### 2.1. Design

The study employed a randomized controlled trial to randomly assign middle school students to two groups, intervention and control groups. In the intervention group, students received four kinds of interventions for 8 weeks based on TPB and SCT, while students in the control group did not receive any interventions apart from pre- and post-measurements like the intervention group. This study lasted 11 weeks, during which the active intervention and comparison period was 8 weeks, while recruitment, screening, and pre- and post-measurement took up the remaining 3 weeks. In detail, the first week was used to screen for and recruit suitable subjects. Then, during the second week, all students finished the questionnaires with regard to psychological and behavioral measurement and wore an ActiGraph accelerometer (model wGT3X-BT) for 7 days at baseline. Finally, in week 11, the post-test procedure was identical to the baseline procedure after the 8-week active intervention period.

### 2.2. Participants

**Figure 1:**
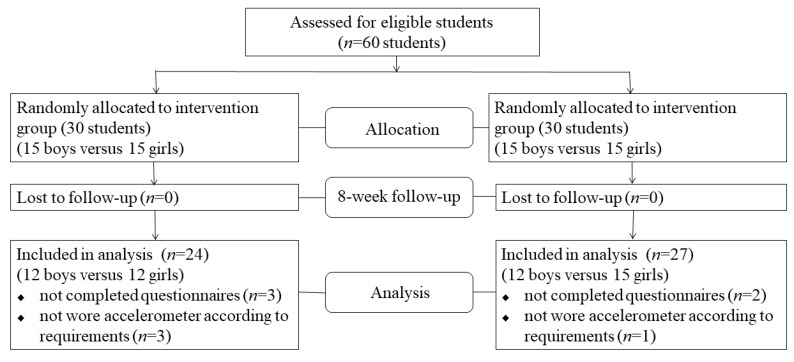
Flowchart of study design.

Sixty seventh-grade students, at similar ages and with similar parental educational background and race, were chosen from a middle school in Beijing based on their voluntary participation in this study. This is a comprehensive school with students from all over China. All students selected were of Han nationality, the most common race in China, and more than 80% of one of their guardians once received higher education. Before the study, the demographic information of these students was collected, then we used a computer randomization application to allocate them to the intervention and control groups. A total of 60 students received the pre-test at baseline, and no one was lost to follow-up at the post-test. However, in the intervention group, 3 participants did not complete all of the psychological questionnaire and 3 participants failed to wear the accelerometer for 3 days according to the requirements. Likewise, at the post-test, 3 participants in the control group did not meet the criteria (detailed criteria can be seen in Section 2.4). Hence, a total of 24 subjects of the intervention group (12 boys and 12 girls) and 27 subjects of the control group (12 boys and 15 girls) were analyzed via screening and cleaning data for questionnaires and ActiGraph. The effective recovery rate of data is 85% (Figure 1).

### 2.3. Intervention

The intervention group received the informational intervention for 8 weeks based on the extended theory of planned behavior, including self-efficacy and outcome expectancy, which are both core concepts of SCT. The active interventions and measurements were mainly conducted by two well-trained assistants (M.Y. and Y.Y.) from our lab team. The main contents of the 8-week intervention were assigned once a week for 45 min, with all the adolescents in the intervention group engaging every time. The main intervention included 5 indoor courses and lectures held in the classroom and 3 outdoor basketball matches on the playground. Participants were also encouraged to make daily exercise plans for 1 week and keep a daily exercise log (recording whether daily exercise was done or not done and some feelings) and submitted the summary every week. Moreover, the head teacher of the intervention group was asked to make a poster about exercise and health with the students. Although the students’ parents did not attend the indoor lectures or outdoor exercises during the intervention period, they were provided with a handbook on exercise and nutrition for adolescents and asked to help their children to keep the exercise log. Also, the PE teachers were invited to help maintain order and ensure safety in the outdoor basketball classes for the intervention group. The detailed contents and descriptions of interventions are as follows and are shown in Table 1.

Module 1: Conduct Indoor Courses and Lectures on Exercise and Health. A series of courses and lectures were taught separately in 5 classes, considering the constructs of TPB and SCT with teenage traits. Specifically, courses on the benefits of exercise, the disadvantages of sedentary behavior, and sportsmanship were assigned to change students’ attitudes toward physical activity. Furthermore, a course on self-recognition and evaluation of adolescent growth and development was developed to improve the students’ outcome expectancy. Additionally, courses on time and health regulation, adolescent obesity and nutritional problems, and practical sports skills guidance were taught in order to enhance students’ PBC and social support from friends, and then advance their SE with regard to physical activity.

Module 2: Write Down Physical Activity Plans. Students were encouraged to make exercise plans every week during the 8 weeks and record the completed plans in time. Also, they were asked to find out the factors that hindered exercise before making new plans the next week and summarize their efforts to overcome obstacles. The aim of this part was to help students further promote EA and EI.

Module 3: Develop Outdoor Sports Matches. Three group basketball matches were assigned in 3 weeks without highly difficult technical requirements. The target of this module was to help students perceive the pleasure and team spirit of physical activity in order to improve their SN, PBC, EI, and SE to a large extent.

Module 4: Seek Social Support from Family and School. In this module, parents were given handbooks on exercise and diet, nutrition, and health to stimulate support for their children. Meanwhile, the head teacher of the intervention group was asked to create a poster about exercise and health in the class so that students would perceive support from school and then increase OE.

Comparison Group. The comparison group followed their school’s scheduled arrangements as usual. That is to say, participants in the control group were not asked to make any changes to their normal school day beyond taking the pre- and post-tests of the PA-related psychological questionnaire, International Physical Activity Rating Scale, and ActiGraph accelerometer, which were provided to both groups at pre- and post-test data collection.

### 2.4. Measures

In addition to demographic information, pre- and post-test questionnaires were obtained to measure extended TPB. In detail, exercise attitude, subjective norms, perceived behavioral control, and intention, which are constructs of TPB, were initially selected according to the TPB survey [20]. The theory of planned behavior is a prominent framework put forward by Ajzen for predicting behavior in various domains and has been increasingly used for instruction of behavioral change interventions. According to the TPB, adolescents with strong exercise intentions, which are thought to be directly impacted by exercise attitude, subjective norms, and perceived behavioral control, are more likely to form healthy PA behavior than adolescents with weaker intentions [20]. All questionnaires assessing the constructs of TPB were made according to the recommended TPB questionnaire manual [30], then we made a few small item description modifications to help adolescents understand in reference to previously validated studies [31,32]. Constructs related to self-efficacy and outcome expectancy were measured in reference to Schwarzer’s study [33], which was an application of Bandura’s study and validated the original SCT survey [21]. PA was recorded in subjective and objective ways for all students. In terms of subjective PA, participants filled out the International Physical Activity Rating Scale (PAS), which was previously developed and verified [34,35], and ActiGraph accelerometers (Model: wGT3X-BT) were used to measure objective PA.

Demographics: Prior to the active intervention, all participants were asked to complete a demographic questionnaire after school with the help of their parents documenting their age, gender, height (cm), weight (kg), their parents’ educational level (e.g., bachelor, master), the number of children in the family, and daily favorite physical activities (e.g., table tennis, swimming, running).

Physical Activity: Subjective PA used 3 items according to PAS to assess students’ usual exercise intensity (e.g., moderate exercise such as prolonged bike riding and table tennis), duration of each activity (e.g., 21 to 30 min), and exercise frequency (e.g., 3–5 days/week). The subjective score for total physical activity (TPA) is the product of exercise intensity, duration, and frequency (Cronbach’s α = 0.82). In addition, every participant was given an ActiGraph accelerometer (model wGT3X-BT) to monitor 7 days of energy consumption (EC), percentage of time spent in sedentary behavior (SB%), and light, moderate, and vigorous intensity of PA (Light-PA%, Moderate-PA%, Vigorous-PA%). Participants were instructed to wear the monitor 24 h a day for 7 consecutive days, except in situations when they would get wet, such as taking a shower. All students were asked to record the time when they wore and took off the device, if they took it off at other unexpected times. The criteria were that participants should wear the ActiGraph for a minimum of 3 valid days/week (at least 2 valid days on weekdays and 1 day on weekends) and have 10 h of wearing time/day. If participants did not meet the criteria, their data were eliminated.

Exercise Attitude: Eight items were adapted to assess attitudes toward planning exercise based on the TPB questionnaire manual [30]. Items were rated on a scale of 1 (strongly disagree) to 5 (strongly agree) and included: “I want to spend time on physical activity,” “I like doing physical activity every day,” and so on. The attitude score was then summed for each item, ranging from 8 to 40, with a higher score meaning a better attitude toward physical activity planning. The items showed acceptable internal reliability (Cronbach’s α = 0.83).

Subjective norms: Seven items were adapted to assess SN toward planning for physical activity, referring to the TPB questionnaire manual [30]. These items were measured from strongly disagree (1) to strongly agree (5) and then summed for subjective norms scores ranging from 7 to 35, where a higher score means a larger influence from others. A sample item for subjective norms is “I participate in physical activities because my friends do it as well.” All items were internally reliable (Cronbach’s α = 0.84).

Perceived behavioral control: Eight items were adapted to assess PBC of exercise, referring to the TPB questionnaire manual and previous studies [30,31,32]. A phrase “moderate and vigorous” was changed into “breath hard or feel tired” to enhance adolescents’ comprehension. Sample items for perceived behavioral control are “I will insist on participating in physical activity even if the weather is cold” and “It is mostly up to me whether I participate in physical activity that makes me feel tired.” A 5-point Likert scale (1 = strongly disagree to 5 = strongly agree) was used to assess the items, and they showed internal consistency (Cronbach’s α = 0.80).

Exercise Intention: Intention for physical activity was assessed by 8 items according to the TPB questionnaire manual and previous studies [30,31,32]. The phrase “moderate and vigorous” was also translated into “breath hard or feel tired” in this construct. A sample item for intention is “I will try to do physical activity that makes me feel tired and breath hard.” A 5-point Likert scale (1 = strongly disagree to 5 = strongly agree) was used to measure these items, which showed internal consistency (Cronbach’s α = 0.84).

Self-efficacy: Exercise self-efficacy was measured with 18 items to evaluate participants’ confidence in doing physical activity and changing PA-related behavior, referring to Bandura and Schwarzer’s study [21,33]. A sample item for self-efficacy is “When I feel stressed, I can still insist on participating in physical activity.” Participants rated each item on a 5-point Likert scale ranging from 1 (completely disagree) to 5 (completely agree), and all items demonstrated internal consistency (Cronbach’s α = 0.863).

Outcome expectancy: Nine items were adapted to assess participants’ expectation of positive outcomes associated with physical activity, in reference to Bandura and Schwarzer’s study [21,33]. A sample item for outcome expectancy is “Physical activity helps me reduce fatigue.” A 5-point Likert scale (1 = strongly disagree to 5 = strongly agree) was used to measure these items, and all items showed internal consistency (Cronbach’s α = 0.89).

### 2.5. Ethical Considerations

The Institutional Review Board of Tsinghua University approved the study (reference 20190093) and all detailed information about the study, such as measurement tools and the intervention period, was plainly provided to all participants and their parents. The opportunity for participants to withdraw from the study at any time was also clearly explained in the informed consent form. A parent or guardian of each of the 60 participants recruited from the seventh grade signed a consent form, and written assent forms were also gathered from the students prior to the active intervention period. All participants were offered Tsinghua University stationery souvenirs and private physical activity assessment as compensation for their participation in baseline and follow-up measurements.

### 2.6. Data Analysis

Data were screened for uncompleted questionnaires and wearing ActiGraph less than 3 days/week (2 days on weekdays and 1 day on weekends) and 10 h/day. The data of height (cm) and weight (kg) for each student was used to calculate body mass index (BMI, kg/m^2^) to indicate body shape, according to the formula 10,000 × weight (kg)/height^2^ (cm). Each subjectively psychological and behavioral variable was computed by related questions. Descriptive analysis was then used, expressed as means and standard deviations (SDs) (95% confidence interval). Moreover, independent *t*-tests were conducted to compare psychological and behavioral differences between intervention and control groups at baseline and follow-up, while paired sample *t*-tests were used to make within-group comparisons after the intervention period for each group. To determine whether changes in psychological and behavioral indices differed for the two groups, repeated measures analysis of variance (ANOVA) was also used to analyze the effect of treatment (intervention versus control group), time (baseline versus end of 8-week intervention), and treatment × time (interaction). All statistical analyses were conducted with IBM SPSS Statistics version 20.0, and *p*-value < 0.05 was considered significant in all tests. Effect sizes for mean differences were expressed as Cohen’s d (difference in means divided by standard deviation of the difference) with values of 0.2, 0.5, and 0.8 denoting small, medium, and large effect sizes, respectively [36].

## 3. Results

### 3.1. Demographic Analysis

The characteristics of both groups are shown in Table 2. This study involved participants (*n* = 51) 12 to 13 years old (mean (M) = 12.12; standard deviation (SD) = 0.33), almost half of whom were boys (47.1%). There were no significant differences in gender distribution, age, weight, height, BMI, number of children, and parents’ educational background between the intervention and control groups, which means that the samples were comparatively balanced. Favorite and regular physical activities between the intervention and control groups were similar and cycling and badminton were the most popular activities among these adolescents.

### 3.2. Psychological Indices of Exercise

**Figure 2:**
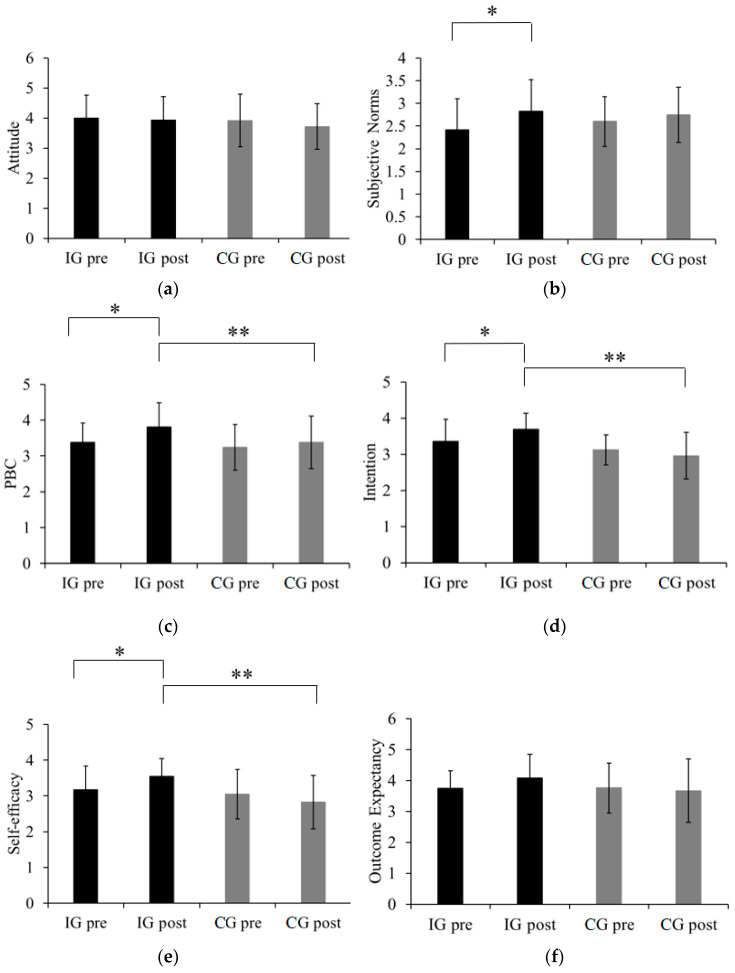
Changes of subjective scores of (**a**) attitude, (**b**) subjective norms, (**c**) perceived behavioral control, (**d**) intention, (**e**) self-efficacy, and (**f**) outcome expectancy regarding physical activity between the two groups from pre-test to post-test. IG pre, pre-test of intervention group; IG post, post-test of intervention group; CG pre, pre-test of control group; CG post, post-test of control group. * Significant within-group difference from pre-test to post-test (*p* < 0.05). ** Significant between-group difference at baseline and after intervention (*p* < 0.05).

Repeated measures ANOVA illustrates the comparison of PA psychological indices between the intervention and control groups, shown in Figure 2. The results show a significant main effect of time in SN (F(1,49) = 8.147, *p* = 0.006, *η_p_*^2^ = 0.143) and PBC (F(1,49) = 17.139, *p* < 0.001, *η_p_*^2^ = 0.259), and a significant main effect of treatment in EI (F(1,49) = 8.945, *p* = 0.004, *η_p_*^2^ = 0.154) and SE (F(1,49) = 4.986, *p* = 0.030, *η_p_*^2^ = 0.092). However, the main effect on PBC (F(1,49) = 4.828, *p* = 0.033, *η_p_*^2^ = 0.090), EI (F(1,49) = 4.226, *p* = 0.045, *η_p_*^2^ = 0.079), and SE (F(1,49) = 4.861, *p* = 0.032, *η_p_*^2^ = 0.090) was qualified by a significant time × treatment interaction. Furthermore, independent *t*-tests were employed to investigate the difference between two groups at baseline and after the intervention, while paired sample *t*-tests were used to compare the change difference of each group over the 8-week period (Table 3). All psychological indices showed no significant difference between the intervention and control groups at baseline. Results indicated a significant increase in score of approximately 0.4 in SN (*p* = 0.041, Cohen’s d = 0.62), PBC (*p* = 0.023, Cohen’s d = 0.72), EI (*p* = 0.043, Cohen’s d = 0.61), and SE (*p* = 0.035, Cohen’s d = 1.36) from pre-test to post-test for the intervention group. SE showed the largest effect size. However, no significant changes were observed in any psychological indices after the intervention period for the control group. After the 8-week intervention, participants in the intervention group showed significantly higher performance in PBC (*p* = 0.032, Cohen’s d = 0.62), EI (*p* < 0.001, Cohen’s d = 1.32), and SE (*p* < 0.001, Cohen’s d = 1.15) related to physical activity compared with the control group.

### 3.3. Behavioral Indices of Exercise

Differences in subjective and objective PA behavioral indices between the intervention and control groups at baseline and at the end of the 8-week intervention are illustrated in Table 4. Exercise duration, frequency, intensity, and total physical activity (TPA) scores represent the subjective PA results, while energy consumption (EC) and percentage of time spent on sedentary behavior (SB%), light-PA, moderate-PA, and vigorous-PA (Light-PA%, Moderate-PA%, and Vigorous-PA%) represent objective results. For subjective results, a significant main effect of time on exercise frequency (F(1,49) = 36.499, *p* < 0.001, *η_p_*^2^ = 0.427) was observed, while a significant effect of time × treatment interaction was shown in exercise intensity (F(1,49) = 6.907, *p* = 0.011, *η_p_*^2^ = 0.124) and TPA (F(1,49) = 2.997, *p* = 0.020, *η_p_*^2^ = 0.09) for the intervention group. Moreover, for objective PA indices, results show a significant main effect of time and treatment in SB% (time: F(1,49) = 9.992, *p* = 0.003, *η_p_*^2^ = 0.169; treatment: F(1,49) = 4.430, *p* = 0.040, *η_p_*^2^ = 0.083) and Light-PA% (time: F(1,49) = 4.317, *p* = 0.043, *η_p_*^2^ = 0.081; treatment: F(1,49) = 8.926, *p* = 0.004, *η_p_*^2^ = 0.154), but the main effect on SB% was constrained by a significant time × treatment interaction (F(1,49) = 6.964, *p* = 0.011, *η_p_*^2^ = 0.124). Furthermore, as the paired sample *t*-tests show in Table 3, even if the values for subjective exercise duration decreased to some extent for both the intervention group (pre = 3.29 ± 1.23, post = 2.71 ± 1.04) and the control group (pre = 2.70 ± 1.10, post = 2.30 ± 0.95), exercise frequency (*p* < 0.001, Cohen’s d = 1.25) and intensity (*p* = 0.028, Cohen’s d = 0.68) showed a significant increase from pre-test to post-test and then a dramatic increase of about 30% for TPA without reaching the significance level (*p* = 0.194; Cohen’s d = 0.38) for the intervention group. Also, a significant decrease of SB% (*p* < 0.001, Cohen’s d = 1.17) was observed, while Light-PA% (*p* = 0.031, Cohen’s d = 0.68) showed a significant upward trend in the intervention group after the 8-week period. After the intervention, participants in the intervention group showed significantly better performance in TPA (40.75 ± 20.09 versus 28.48 ± 14.63; *p* = 0.015, Cohen’s d = 0.72), including more Light-PA% (*p* < 0.001) and less SB% (*p* < 0.001) than the control group, with a large effect size of 1.12 and 1.22, respectively.

## 4. Discussion

The focus of the current study was to investigate whether a multicomponent intervention based on the extended TPB combined with self-efficacy and outcome expectancy could improve physical activity and positively change PA-related psychological effects among Chinese adolescents for a period of eight weeks. The hypothesis was largely supported, as the intervention group, according to the pre- and post-test, showed significant improvement of SN, PBC, EI, SE, SB%, and exercise frequency and intensity, especially in Light-PA%. On the other hand, there were no significant changes in EA and OE or other behavioral variables, such as exercise duration and moderate to vigorous PA. As a result, our study is in line with previous intervention studies [27,37,38,39] indicating that such short-term school and theory-based intervention has significantly positive effects on PA-related psychological constructs and behavioral changes. Overall, the specific intervention was effective in facilitating behavioral and psychological outcomes highly related to physical activity and health among Chinese adolescents in eight weeks.

The results indicate that physical activity in the intervention group significantly increased across the specific intervention, whereas SB% dramatically decreased. However, a downward trend of subjective exercise duration was observed in both intervention and control groups, even if the intervention group showed a significant increase in subjective exercise frequency and intensity, finally resulting in increased physical activity. This finding can be understood against the background of seasonal changes and the final exam period. Considering the baseline test was at the beginning of the semester while the follow-up one was near final exams and the weather in that period was getting colder daily, all participants had adequate reason to exercise for a shorter time. However, the positive change of exercise frequency and intensity and decreased sedentary behavior in the intervention group still verifies the success of this intervention modality, in agreement with previous studies [39,40]. When compared with an intervention study conducted at home [41] that did not find any significant PA change after intervention, this school-based intervention study showed better effects. In addition, the intervention group’s TPA measured subjectively showed a significant increase compared with the control group, but total energy consumption measured with the ActiGraph over seven days did not show a significant change. This difference might be because the short-term intervention lasted for only eight weeks. According to the increasing rate and effect size of EC, a longer intervention might lead to a more significant change of PA behavior [26].

Furthermore, it is worth mentioning that there was significant change in the level of Light-PA%, but moderate and vigorous PA did not substantially change over the intervention. This interesting finding can be attributed to two main reasons. First, although most previous studies recommended focusing on increasing MVPA [41,42], it might not be an easy task to facilitate adolescents’ MVPA in the short-term with our intervention, which can only be conducted 45 min once a week for eight weeks. After all, in order to improve adolescent boys’ MVPA, Robbins [43] conducted a similar intervention, consisting of an extra 90 min after-school physical activity club four days per week, while the intervention program assigned by Sutherland [26] lasted for at least one year. Another reason might be that the main types of interventions, including group basketball matches and exercise and health courses, were implemented in order to raise students’ interest in exercise, awareness, and participation rate. Therefore, participants might concentrate on not doing the exercise, but enjoy the happiness and spirit from team games. As a result, the actual intensity results of the intervention might be at a low level. For these reasons, Chinese adolescents in this study might prefer an intensity of physical activity such that they do not feel physically burdened but receive satisfaction from it and then gradually form exercise habits on their own.

The results also indicate that SN, PBC, SE, and EI were positively changed over the 8-week intervention, and this finding was supported by previous studies [27,39,44,45]. It is well documented that SE, which can be a direct or indirect determinant of initiating and maintaining physical activity [46], is the most important construct of SCT. However, it is a difficult task to improve self-efficacy, and the most efficient way to do it is to take part in actual physical activity personally and persistently [21,47]. Lee’s study [28], which was designed based on the theory of self-efficacy without any practical PA instruction, found no significant promotion in SE among female students owing to the high pressure of final examinations. In contrast, the specific intervention in this study based on the combined TPB and SCT can significantly improve self-efficacy and physical activity due to the multiple and interrelated components. Also, SN were regarded as an extremely difficult construct to positively change in previous intervention programs [45], and a lower predicted variance of that could be found in a related theoretical study [48]. In our study, the scores of subjective norms in the two groups were apparently lower than in other psychological constructs, which means most participants did not regard their social network as being supportive of exercise behavior. However, because of the multiple modules designed in our intervention program aimed at improving adolescents’ SN, participants in the intervention group showed a significant increase of that over time, with a medium effect size.

Also, the psychological effect of PBC increased significantly, mainly because of the thoughtful consideration of different exercise ability, interests, and purpose of the Chinese adolescent students. In detail, we first emphasized the importance of PA participation and mastering basic exercise skills for students with little exercise background, and also helped them to seek their value and pleasure in group basketball matches, and then we recommended that students with more exercise experience do more skillful after-class physical activity. Hence, participants in the intervention group raised their PBC significantly, with a large effect size. Moreover, EI, influenced by PBC, EA, and SN, is the strongest predictor that impacts actual exercise behavior. Thanks to the improved PBC and SN during the intervention period, exercise intention was significantly promoted, similar to previous research [49].

Nevertheless, the other two psychological variables, EA and OE, did not show a significant increase after the intervention. The pressure from final exams and the influence of cold weather may account for the nonsignificant change of attitude, but luckily, across the period of the study, both intervention and control participants similarly reported very positive attitudes toward engaging in physical activity. Furthermore, the medium effect size suggests that a longer intervention may in fact contribute to further significant differences. An improved intervention is needed in a further study to seek more support from family, teachers, friends, and schools.

There are a number of limitations that should be addressed for future research. The first limitation is related to the sample size. Considering the cost and operability of objective PA measures via ActiGraph, we did not choose a bigger sample size for the intervention and control groups, even if we were to use a randomized controlled trial. This study presents a series of preliminary results of the specific intervention, and it may be better to increase the sample size for further PA intervention exploration in China. The second limitation is related to the characteristics of the school selected. We only chose adolescents from one school in Beijing, and it was not representative enough of the whole Chinese adolescent population. Although this selected school is comprehensive, with students from all over the country, and can represent a large number of Chinese adolescents, more intervention studies need to be done in other schools in the future. The third limitation is related to the intervention period. Referring to a previous PA intervention study, 8 weeks was enough for us to carry out all intervention modules and influence students’ PA-related psychological and behavioral effects preliminarily. However, cognitive behavioral change is a relatively slow process from the internal world to outward appearance, and many Chinese adolescents have 16 weeks of PE lessons in a semester. So, if possible, it may be better to extend the intervention period and integrate it with the school syllabus, to determine whether the positive changes observed are sustained, and the total PA energy consumption may increase significantly with long-term follow-up according to the tendency of this study.

## 5. Conclusions

The strength of the present study is that it conducted a multicomponent intervention based on the extended theory of planned behavior combined with self-efficacy and outcome expectancy for unstudied Chinese adolescents using a longitudinal study design, because most of the related research has primarily been carried out in Western societies. In this study, the specific intervention combining psychological and behavioral strategies positively changed PA-related psychological variables such as self-efficacy and significantly contributed to decreased sedentary behavior and increased physical activity, especially exercise frequency and light-intensity PA, among Chinese adolescents. The retention rate in the current study was quite high (85%) across the 8-week intervention, which is indirect evidence supporting the Chinese adolescents’ receptivity to this intervention modality integrating PA-related behavioral and psychological strategies. This preliminary study supports comprehensive intervention integrating mental and physical strategies in non-Western adolescents. Therefore, even if this is a pilot study and there are some limitations, this creative study suggests that it is vital to consider not only physical activity behavior itself, but also the various PA-related psychological factors with appropriate health-promotion theories as the basis when planning and implementing intervention programs for Chinese adolescents. This kind of intervention has the potential to form and facilitate spontaneous physical activity, and more intervention studies, with longer periods and bigger sample sizes, should be done in the future for different kinds of Chinese adolescents.

## Figures and Tables

**Table 1:** Details of school-based physical activity intervention for seventh-grade adolescents.

Week	Module	Content	Position	Companion	Main Target
1	1	Lecture on benefits of exercise, disadvantages of sedentary behavior, and sportsmanship, 45 min, taught by M.Y.	Classroom	None	EA
2	1	Course on self-recognition and evaluation of adolescent growth and development, 45 min, taught by Y.Y.	Classroom	None	OE
3	1	Lecture on health regulation, 45 min, taught by Y.Y.	Classroom	None	PBC, SN, SE
4	1	Lecture on adolescent obesity and nutrition problems, 45 min, taught by M.Y.	Classroom	None	PBC, SN, SE
5	1	Course on practical sports skills guidance, 45 min, taught by M.Y.	Classroom	None	SE
6	3	First course on basic basketball skills and basketball match, 45 min, taught by Y.Y.	Playground	None, but with PE teacher to ensure safety	PBC, SN, SE, EI
7	3	Second course on basic basketball skills and basketball match, 45 min, taught by Y.Y.	Playground	None, but with PE teacher to ensure safety	PBC, SN, SE, EI
8	3	Third course on basic basketball skills and basketball match, 45 min, taught by M.Y.	Playground	None, but with PE teacher to ensure safety	PBC, SN, SE, EI
1–8	2	Students encouraged to make exercise plan every week and keep exercise log every day, then submit log summary every week.	At home	At least one parent	EI, EA
2–5	4	Students encouraged to discuss lectures on exercise and health with friends and make posters with head teacher around related themes.	Classroom or home	Head teacher and friends	OE

EA, exercise attitude; OE, outcome expectancy; PBC, perceived behavioral control; SN, subjective norm; SE, self-efficacy; EI, exercise intention; PE, physical education.

**Table 2:** Demographics of participants at baseline (*n* = 51).

Variable	Intervention Group (*n* = 24)	Control Group (*n* = 27)	*χ*^2^(*p*)/*t*(*p*)
Gender (female/male)	12/12	15/12	0.16 (0.69)
Age (years)	12.08 ± 0.30	12.15 ± 0.40	0.72 (0.48)
Body weight (kg)	49.29 ± 8.51	48.48 ± 10.23	−0.31 (0.76)
Height (cm)	161.25 ± 7.30	160.52 ± 6.31	−0.40 (0.69)
BMI (kg/m^2^)	18.95 ± 3.27	18.78 ± 3.76	−0.18 (0.86)
Only child (yes/no)	16/8	16/11	0.30 (0.59)
Father higher education (yes/no)	20/4	22/5	0.03 (0.86)
Mother higher education (yes/no)	21/3	20/7	1.45 (0.23)
Regular physical activity			
Cycling	10/24	13/27	
Table tennis	4/24	5/27	
Badminton	13/24	13/27	
Jogging/running	6/24	9/27	
Basketball	6/24	6/27	
Skipping rope	3/24	4/27	
Swimming	5/24	5/24	

Description of participants at study baseline, including common demographics, and most regular and favorite physical activities of participants. BMI: body mass index, calculated by the formula 10,000 × weight (kg)/height^2^ (cm). Father/mother higher education means father/mother of participant once received higher education.

**Table 3:** Results of psychological indices between the two groups at baseline and follow-up.

Variables	Intervention Group (*n* = 24)		Control Group (*n* = 27)	
	Pre-Test Mean ± SD	Post-Test Mean ± SD	Pre-Test Mean ± SD	Post-Test Mean ± SD
EA	3.99 ± 0.78	3.93 ± 0.79	3.92 ± 0.88	3.72 ± 0.76
SN	2.41 ± 0.69	2.83 ± 0.69 *	2.60 ± 0.55	2.75 ± 0.61
PBC	3.38 ± 0.54	3.81 ± 0.68 * ¶	3.24 ± 0.64	3.38 ± 0.73
EI	3.36 ± 0.61	3.69 ± 0.45 * ¶	3.12 ± 0.42	2.96 ± 0.65
SE	3.17 ± 0.67	3.55 ± 0.49 * ¶	3.05 ± 0.69	2.83 ± 0.75
OE	3.74 ± 0.58	4.08 ± 0.76	3.76 ± 0.81	3.67 ± 1.03

* Significant within-group difference from pre-test to post-test (*p* < 0.05). ¶ Significant between-group difference after intervention (*p* < 0.05). EA, exercise attitude; OE, outcome expectancy; PBC, perceived behavioral control; SN, subjective norms; SE, self-efficacy; EI, exercise intention; SD, standard deviation.

**Table 4:** Results of behavioral indices between the two groups at baseline and follow-up.

Variables	Intervention Group (*n* = 24)		Control Group (*n* = 27)	
	Pre-Test Mean ± SD	Post-Test Mean ± SD	Pre-Test Mean ± SD	Post-Test Mean ± SD
Duration	3.65 ± 1.23	2.71 ± 1.04	2.70 ± 1.10	2.30 ± 0.95
Frequency	2.75 ± 1.07	3.92 ± 0.83 *	3.04 ± 0.98 *	3.70 ± 0.72
Intensity	3.17 ± 1.09	3.83 ± 0.87 * ¶	3.52 ± 0.85	3.33 ± 0.68
TPA	31.96 ± 25.76	40.75 ± 20.09 ¶	31.04 ± 21.15	28.48 ± 14.63
EC	11,362.11 ± 5245.98	13,408.31 ± 5971.38	13,337.84 ± 5335.88	12,545.45 ± 4628.22
SB%	68% ± 10%	58% ± 7% * ¶	68% ± 12%	67% ± 8%
Light-PA%	22% ± 7%	25% ± 4% * ¶	19% ± 7%	20% ± 5%
Moderate-PA%	5% ± 2%	6% ± 1%	6% ± 2%	6% ± 1%
Vigorous-PA%	6% ± 3%	6% ± 2%	7% ± 3%	6% ± 2%

* Significant within-group difference from pre-test to post-test (*p* < 0.05). ¶ Significant between-group difference after intervention (*p* < 0.05). Duration, frequency, and intensity represent subjective scores of exercise duration per time, exercise frequency per week, and exercise intensity per time. TPA, total physical activity, represents product scores of exercise duration, frequency, and intensity. EC, energy consumption, represents totally objective energy consumed in physical activity in seven days. SB%, light-PA%, moderate-PA%, and vigorous-PA% represent objective percentages of time spent on sedentary behavior and light, moderate, and vigorous physical activity in seven days.
